# Numerical Study on Hydrodynamic Characteristics and Electrochemical Performance of Alkaline Water Electrolyzer by Micro-Nano Surface Electrode

**DOI:** 10.3390/ma15144927

**Published:** 2022-07-15

**Authors:** Ye Xia, Mengyu Gao, Jincheng Yu, Yang Si, Laijun Chen, Shengwei Mei

**Affiliations:** 1 New Energy (Photovoltaic) Industry Research Center, Qinghai University, Xining 810016, China; 18081291358@163.com (Y.X.); yjc18636285020@163.com (J.Y.); chenlaijun@tsinghua.edu.cn (L.C.); meishengwei@tsinghua.edu.cn (S.M.); 2 State Key Laboratory of Control and Simulation of Power System and Power Generation Equipment, Electric Machinery Department, Tsinghua University, Beijing 100084, China

**Keywords:** alkaline water electrolysis, two-phase flow, micro-nano fabrication, micro-nano surface electrode, numerical simulation

## Abstract

This study constructed a two-dimensional alkaline water electrolyzer model based on the two-phase flow Euler–Euler model. In the model, the micro-nano surface electrodes with different structure types and graphic parameters (distance, height, and width) were used and compared with the vertical flat electrode to evaluate their influence on electrolysis performance. The simulation results show that the performance of the micro-nano surface electrode is much better than that of the vertical flat electrode. The total length of micro-nano structural units relates to the contact area between the electrode and the electrolyte and affects the cell voltage, overpotential, and void fraction. When rectangular structural units with a distance, height, and width of 0.5 µm, 0.5 µm, and 1 µm are used, the total length of the corresponding micro-nano surface electrode is three times that of the vertical flat electrode, and the cathode overpotential decreases by 65.31% and the void fraction increases by 54.53% when it replaces the vertical flat electrode.

## 1. Introduction

Increased demand for energy, depletion of fossil fuels, and global warming have become global challenges recently; thus, there is an urgent need for renewable energy development [1]. Among all the alternatives to fossil fuels, hydrogen is one of the most promising clean energies due to its abundant reserves and high energy density [2]. Hydrogen renewable energy system includes hydrogen production, storage, and utilization [3]. In the process of hydrogen production, high-purity hydrogen produced by water electrolysis is favored by hydrogen-based fuel cells [4]. Water electrolysis is divided into alkaline water electrolysis, proton exchange membrane, and solid oxide electrolysis [5,6]. Hydrogen production from alkaline water electrolysis is closest to industrial application, and a traditional alkaline water electrolyzer (AWE) mainly includes a cathode, anode, diaphragm, and electrolyte. Among them, the activation energy barrier and catalytic active area of the electrode determine the electrolysis efficiency [7,8,9,10].

Experimentally, as early as 1982, researchers realized that the surface roughness of an alloy catalyst is higher than that of pure metal and has more catalytic active sites. Brown et al. [11] prepared the electrodes with a low overvoltage for hydrogen evolution and good stability. The electrodes with a larger catalytic surface area can reduce the overvoltage in the 70–90 mV range at 70 °C and the current density of 1 A cm^−2^. In 2020, Zhang et al. [12] synthesized Ni_3_S_2_ three-dimensional rod array structures doped with trace amounts of Fe. Experiments showed that the catalyst has excellent catalytic activity and corrosion resistance in acidic, neutral, and alkaline solutions. In the 1.0 M KOH solution, only small overpotentials of 105 mV and 213 mV were required to achieve the current density of 10 mA cm^−2^ for hydrogen evolution reaction (HER) and oxygen evolution reaction (OER), respectively. In 2021, Wang et al. [13] synthesized an IrNi-FeNi_3_ hybrid self-supported on nickel foam with unique nanosheets, and the results showed that only 1.47 V is required for overall water splitting to arrive at 10 mA cm^−2^. They found that the unique self-supported nanosheets structure on nickel foam can expose more active sites, and the hybridization of IrNi with FeNi_3_ can promote the intrinsic activity of catalyst. According to the above, it can note that increasing the catalytic surface area can reduce the overpotential of hydrogen production in actual experiments.

In recent years, many numerical studies have been carried out on AWE modeling, characterization, and analysis, mainly accomplished by coupling the current distribution model and computational fluid dynamics (CFD) model [14]. Rodríguez et al. [15] established a two-dimensional (2D) CFD model of AWE, which considered and analyzed the electrochemical phenomena and fluid dynamics involved in AWE under different operating conditions and verified them by experiments. For all cases, the model fitted very well with the experimental data, and the error of the polarization curve was less than 1%. Zarghami et al. [16] used the Euler–Euler model to simulate multiphase flow in AWE. Their simulation results compared with the experimental data and were in reasonable agreement with the experimental data at a higher current density due to the turbulence dispersion force added. Liu et al. [17] constructed a two-phase bubble flow 2D numerical model in the electrolytic cell to study hydrodynamics and mass transfer based on the Euler–Euler method. The results showed that the effects of different operating conditions on the volume fraction and bubble layer were in good agreement with the experimental results. According to the above, it can note that with the development of computer science, the numerical simulation of hydrogen production by water electrolysis has gradually become a research hotspot [18,19,20]. Compared with the actual experiment, one of the advantages of numerical simulation is that it can get relevant results without actual experiments, which is cost-effective and has a low time cost for experimental design.

As mentioned above, increasing the catalytic surface area is an important method to improve hydrogen production efficiency by water electrolysis [21,22]. Meanwhile, surface patterning is essential to improve the surface area of materials [23,24,25]. Therefore, it is a crucial way to design the electrode surface to obtain the influence rule of pattern parameters on the efficiency of hydrogen production by water electrolysis through numerical simulation, especially for “top-down” construction approaches such as lithography [26,27], which can reduce its design cost. However, there are few simulation studies on the influence of micro-nano structure graphic parameters on hydrogen production by water electrolysis. Therefore, this study numerically investigates the impact of micro-nano structures on the electrochemical and fluid performance of AWE based on the Euler–Euler method. The influence factors of the micro-nano structure were added under the steady-state, 2D, and two-phase flow CFD model considering turbulence.

## 2. Model

### 2.1. Physical Model

**Figure 1:**
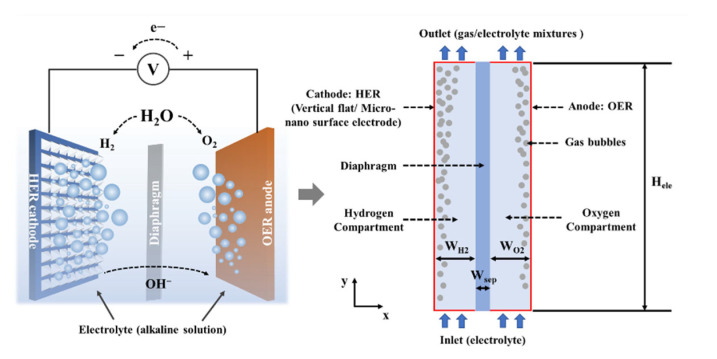
Schematic diagram of the 2D geometric model of AWE.

The 2D geometric model of the AWE referring to commercial AWE is shown in Figure 1 [28,29]. The AWE is divided into cathodic and anodic compartments by a separator known as a diaphragm, which is to avoid the mixing of the two gases and maintains a low resistivity. These compartments are submerged in the electrolyte, an aqueous potassium hydroxide (KOH) solution. The cathode and the anode are located on the left and right walls of the gas compartment, respectively. The hydrogen gas (H_2_) evolves at the cathode, whereas oxygen gas (O_2_) is generated at the anode. The electrochemical reactions are shown in Equation (1) and Equation (2), respectively.

**Figure 2:**
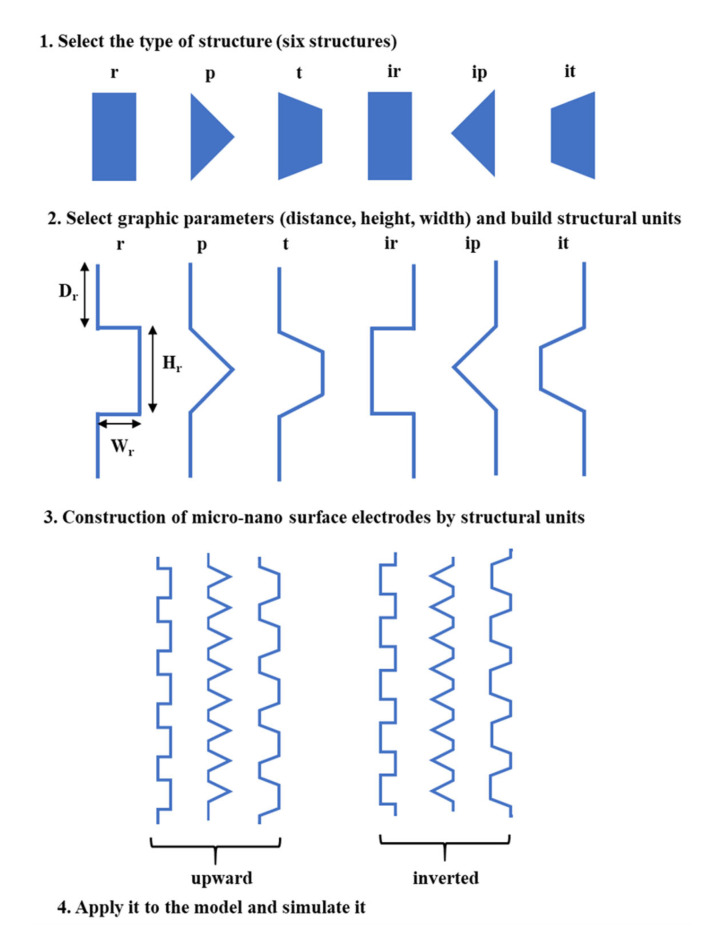
Design flow of micro-nano surface electrodes.

For the cathode, a vertical flat electrode was changed into a micro-nano surface electrode to investigate the influence of micro-nano structures on the AWE performance in this study. The design flow of micro-nano surface electrodes is shown in Figure 2. Firstly, six structures are mainly selected, namely rectangular (r), triangle (p), trapezoidal (t), inverted rectangular (ir), inverted triangle (ip), and inverted trapezoidal (it). Then the graphic parameters (distance, height, width) are selected to construct the structural units. Thirdly, the micro-nano surface electrodes are formed. Finally, the micro-nano surface electrodes are applied to the AWE model and simulated.

The electrode surfaces are set at the height of 10 mm, the same as the diaphragm. The diaphragm is a thin rectangle with a width of 1 mm. The cathodic and anodic compartments are described as thick rectangles with a width of 2 mm. The KOH electrolyte enters from the inlet, and H_2_ and O_2_ bubbles evolve on the cathode and anode, respectively. The generated bubbles move along the *x* and *y* axes to form a bubble layer, and the resulting two-phase mixture (gas and electrolyte) leaves from the outlet.

(1) $${2H}_{2}{O(l)+2e}^{-}{=H}_{2}{(g)+2\mathrm{OH}}^{-}$$

(2) $${4\mathrm{OH}}^{-}{=2H}_{2}{O(l)+O}_{2}{(g)+4e}^{-}$$

### 2.2. Numerical Model

Multiphase flow models are broadly classified into Euler–Euler and Euler–Lagrange approaches. The Euler–Lagrange model is challenging to deal with in the large number of bubbles that consume a long computing time. Moreover, the Euler–Lagrange model generally does not consider the bubble–bubble interactions and ignores the “numerical volume” occupied by bubbles [30,31]. Hence, it is not suitable for this study to consider turbulent dispersion force. The Euler–Euler model can handle numerous types of multiphase flow and is suitable for simulating the macroscopic behavior of many bubbles rising through the liquid [32]. Consequently, the Euler–Euler model is generally considered the most suitable for the two-phase flow.

As previously mentioned, the electrochemical phenomena and fluid characteristics of AWE are numerically simulated using COMSOL Multiphysics 5.6. In this study, the model was built using the “water electrolyzer” module to calculate the current distribution and plot the polarization curve. The “Euler–Euler model, turbulent flow” module was used to study the generation and distribution of two gases in compartments. This model was solved in a steady-state.

The model has been designed using the following assumptions to ensure a manageable problem [33,34,35]:

1. The fluids in both phases are Newtonian, viscous, and incompressible;
2. The physical properties remain constant;
3. The two phases share the same pressure field;
4. The electrolyte is considered distributed uniformly due to the distribution of the ions has little effect on the result;
5. The flow is considered isothermal. Thus, heat exchange and energy equation are not considered;
6. The influence of surface tension is negligible;
7. No bubble coalescence or break-up occurs, and the bubble diameter can be considered constant at a given current density;
8. The bubble–bubble interaction is considered by introducing the turbulent dispersion force;
9. Although the Reynolds number is small, the fluid is considered laminar flow. However, considering the observed turbulence phenomenon by Boissonneau et al. [36] and Aldas et al. [37], a simple turbulence model is selected to describe the turbulence behavior in the study;
10. Since alkaline electrolysis is a complex physical problem involving two-phase flow and the construction of micro-nano surface electrodes is complicated, all issues are simplified to 2D and steady-state simulation.

#### 2.2.1. Secondary Current Distribution Model

Secondary current distribution is used to simulate the electrical property of AWE in this work. Equation (3) shows the total potential obtained by simulation [38]. Based on the above assumptions, Ohm’s law, current conservation, and the Butler–Volmer equation are used to solve the current density distribution and explain the influence of electrode kinetics, as shown in Equation (4), Equation (5), and Equation (6), respectively [39,40,41,42].

(3) $$U_{\mathrm{cell}}{=E}_{\mathrm{rev}}{+\eta }_{\mathrm{ohm}}{+\eta }_{\mathrm{act},a}{(j)+\eta }_{\mathrm{act},c}\left(j\right)$$

Ohm’s law:

(4) $$\vec{j}=-\sigma \nabla \varphi$$

Current conservation:

(5) $$\nabla \cdot \vec{j}=Q$$

Butler–Volmer equation:

(6) $$j_{\mathrm{loc}}{=j}_{0}(\exp(\frac{\alpha_{a}F\eta }{\mathrm{RT}})-\exp(-\frac{\alpha_{c}F\eta }{\mathrm{RT}})$$

where $E_{\mathrm{rev}}$ is the reversible voltage in V, $\eta_{\mathrm{ohm}}$ is the ohmic overpotential in V, $\eta_{\mathrm{act},a}$ and $\eta_{\mathrm{act},c}$ is the activation overpotential at anode and cathode respectively in V, $\vec{j}$ is the current density vector in A m^−2^, φ is the electrical potential in V, σ is the conductivity in S m^−1^, Q is a general current source term in A m^−3^, $j_{\mathrm{loc}}$ is the local charge transfer current density in A m^−2^, $j_{0}$ is the exchange current density in A m^−2^, $\alpha_{a}$ and $\alpha_{c}$ are the anodic and cathodic charge transfer coefficient, respectively, η is the activation overpotential in V, F is the Faraday constant in C mol^−1^, and R is the universal gas constant in J mol^−1^ K^−1^.

#### 2.2.2. CFD Model

The Euler–Euler model is applied to represent the two-phase flow behavior. The model calculates the void fraction of each phase in two phases without defining each bubble in detail. Each phase has a velocity field, and the dynamics of each phase are described by the momentum equation and the continuity equation, as shown in Equation (7) and Equation (8), respectively. Moreover, the volume fractions are assumed continuous functions of space and time, and their sum is equal to one, i.e., $\alpha_{g}{+\alpha }_{l}=1$ [34].

Momentum equation:

(7) $$\frac{\partial }{\partial t}{(\rho }_{k}\alpha_{k}{\vec{u}}_{k})+\nabla {(\rho }_{k}\alpha_{k}{\vec{u}}_{k}{\vec{u}}_{k}{)=\alpha }_{k}\rho_{k}\vec{g}-\alpha_{k}\nabla p+\nabla {(\alpha }_{k}\tau_{k}{)+\vec{F}}_{k}$$

Continuity equation:

(8) $$\frac{\partial }{\partial t}{(\alpha }_{g}\rho_{g}{+\alpha }_{l}\rho_{l})+\nabla {(\alpha }_{g}\rho_{g}{\vec{u}}_{g}{+\alpha }_{l}\rho_{l}{\vec{u}}_{l})=0$$

where u is the velocity in m s^−1^, p is the pressure in Pa, α is the void fraction, ρ is the density in kg m^−3^, g is the gravitational acceleration in m s^−2^, τ is the stress tensor in N m^−2^, F_k_ is the volume force in N m^−3^, and the subscript k refers to gas phase (g) or liquid phase (l).

The transport equation of the void fraction of gas is shown in Equation (9).

(9) $$\frac{\partial \alpha_{g}\rho_{g}}{\partial t}+\nabla {(\alpha }_{g}\rho_{g}{\vec{u}}_{g})=-{\dot{m}}_{\mathrm{gl}}$$

where ${\dot{m}}_{\mathrm{gl}}$ is the mass transfer rate from gas to liquid in g m^−3^ s^−^^1^.

In this simplified 2D model, the gas flow generated (g m^−2^ s^−1^) on the active surface of the electrode is defined by the Faraday equation, as shown in Equation (10).

(10) $${\dot{m}}_{g}=\frac{j\cdot M_{g}}{\mathrm{nF}}$$

where j is the current density in A m^−2^, M_g_ is the molar mass of the gas in g mol^−1^, n is the number of transferred electrons, and the subscript g refers to H_2_ or O_2_.

Although the Reynolds number range is low, referring to the turbulence phenomenon observed by Mat et al. [37,43], and to better characterize the fluid flow behavior, therefore, a simple turbulence model was used. For turbulence, the k-ε model is applied. The stress tensor τ and the turbulent viscosity $\mu_{T}$ (Pa s) are shown in Equation (11) and Equation (12), respectively. The k-ε model solves two additional transport equations for two additional variables: the turbulent kinetic energy k (m^2^ s^−2^) and the turbulent dissipation rate ε (m s^−3^), as shown in Equation (13) and Equation (14), respectively [44].

(11) $$\tau_{k}{=(\mu }_{k}{+\mu }_{T})(\nabla {\vec{u}}_{k}+(\nabla {\vec{u}}_{k}{)}^{T}-\frac{2}{3}(\nabla \cdot {\vec{u}}_{k})I)-\frac{2}{3}\rho_{k}\mathrm{kI}$$

(12) $$\mu_{T}{=\rho }_{k}C_{\mu }\frac{k}{\varepsilon }$$

(13) $$\rho \frac{\partial k}{\partial t}+\rho \vec{u}\nabla k=\nabla \cdot ((\mu +\frac{\mu_{T}}{\sigma_{k}})\nabla {k)+P}_{k}-\rho \varepsilon$$

(14) $$\rho \frac{\partial \varepsilon }{\partial t}+\rho \vec{u}\nabla \varepsilon =\nabla ((\mu +\frac{\mu_{T}}{\sigma_{k}})\nabla {\varepsilon )+C}_{\varepsilon ,1}\frac{\varepsilon }{k}P_{k}-C_{\varepsilon ,2}\rho \frac{\varepsilon^{2}}{k}$$

where µ is the viscosity in Pa s, I is the unit tensor in N m^−2^, the subscript k refers to gas phase (g) or liquid phase (l), $C_{\mu }$, $C_{\varepsilon ,1}$, $C_{\varepsilon ,2}$, and $\sigma_{k}$ are all constants in the turbulence model, and $P_{k}$ is related to bubble-induced turbulence.

In addition to the drag force hindering the flow in the AWE, in fact, with the continuous generation and accumulation of bubbles, the collision between bubbles increases, which triggers the diffusion of bubbles from a high concentration to a low concentration. Therefore, based on previous work, drag force and turbulent dispersion force are considered in the model, as shown in Equation (15) and Equation (16), respectively [45,46].

(15) $${\vec{F}}_{D}=-\frac{3}{4}\rho_{k}\alpha_{k}\frac{C_{d}}{d_{b}}\left|{\vec{U}}_{r}\right|{\vec{U}}_{r}$$

(16) $${\vec{F}}_{\mathrm{BD}}=-\alpha_{k}\rho_{k}\frac{K_{g}}{d_{b}}\left|{\vec{U}}_{r}\right|\nabla \alpha_{k}$$

where $C_{d}$ is the drag coefficient, K_g_ is the diffusion factor of mixed bubbles in m s^−2^, and $d_{b}$ is the bubble diameter in m. The drag coefficient is defined by the Schiller–Naumann model as shown in Equation (17).

(17) Cd=24Re(1+0.15Re0.687) Re≤10000.44 Re>1000

where Re is the Reynolds number of bubbles calculated according to relative velocity, Re=ρlU→rdb/μl.

#### 2.2.3. Initial Values and Boundary Conditions

**Figure 3:**
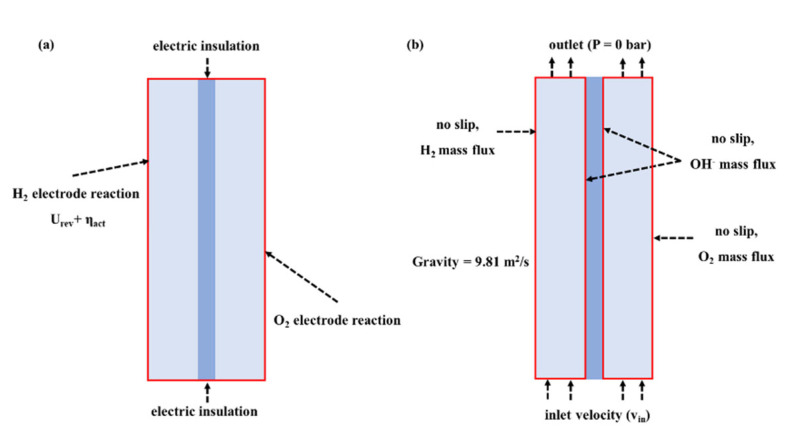
Boundary conditions of AWE. (**a**) Electrochemical model; (**b**) Hydrodynamic model.

The boundary conditions of the electrochemical and hydrodynamic models are shown in Figure 3. For the electrochemical model, the top and bottom boundaries of the model are electric insulation. For the hydrodynamic model, the velocity inlet and pressure outlet are used as the boundary conditions at the inlet and the outlet. No slip is used as the boundary condition of electrodes and the boundaries of the diaphragm. Their velocity is set to 0 m s^−1^. At the electrode surfaces, gas and liquid mass flux boundary conditions are used. At the boundaries of the diaphragm, the liquid phase mass flux corresponding to the ionic flux of OH^−^ from the current distribution model is adopted.

Because of the bubble effect, the conductivity of the KOH electrolyte will decrease, and the generated bubbles will cover the electrode surface, affecting the exchange current density of the electrode reaction. Therefore, the effective electrolyte conductivity and the effective exchange current density are described according to the Bruggeman equation, as shown in Equation (18) and Equation (19), respectively [28,33].

(18) $$\sigma_{\mathrm{eff}}{=\sigma }_{0}{{(1-\alpha }_{g})}^{1.5}$$

(19) $$i_{\mathrm{eff}}{=i}_{0}{{(1-\alpha }_{g})}^{1.5}$$

where $\sigma_{\mathrm{eff}}$ is the effective electrolyte conductivity in S m^−1^, $\sigma_{0}$ is the total electrolyte conductivity in S m^−1^, $i_{\mathrm{eff}}$ is the effective exchange current density in A m^−2^, $i_{0}$ is the exchange current density in A m^−2^, $\alpha_{g}$ is the gas volume fraction, and the subscript g refers to H_2_ or O_2_.

The initial values for solving the model are shown in Table 1.

### 2.3. Grid Study and Validation

In this study, a physics-controlled mesh was used to adopt the modules, and a triangular mesh was generated. Mesh elements were fine predefined on the electrolyte domain and extra fine on the electrodes and diaphragm boundaries to correctly handle the effects in these contours. For details of grid and model study and validation, please refer to Table S1, Figures S1–S3 of Supplementary Materials.

## 3. Results

### 3.1. Vertical Flat Electrode

The bubble size produced in water electrolysis is affected by multiple factors, such as current density, electrode, and electrolyte flow rate. Therefore, two constant bubble diameters are considered in this paper to fit the actual situation as far as possible. According to the literature results [47,48], the bubble diameters produced by the vertical flat electrode and micro-nano surface electrode are 200 µm and 50 µm, respectively. The effects of these two bubble diameters were simulated using the vertical flat electrode to show the influence of bubble diameter change on electrolysis performance.

**Figure 4:**
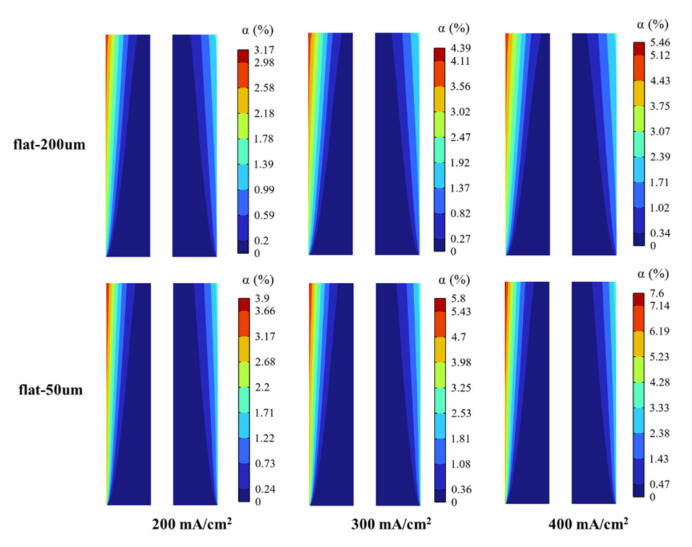
Gas void fraction distribution corresponds to two bubble diameters under different current densities.

The gas void fraction distribution corresponding to two bubble diameters at different current densities is shown in Figure 4. It is seen that the bubble layer is parabolic distribution, and the void fraction increases with the increase of current density because the electrochemical reaction rate is proportional to the current density. With the increase of current density, the gas production rate increases, which leads to the increase of void fraction, thus promoting the thickening of the bubble layer. Moreover, the high void fraction will lead to more turbulent dispersion, which will distance the bubbles far from the electrode, expand the bubble layer, and increase the local void fraction by increasing the total void fraction.

**Figure 5:**
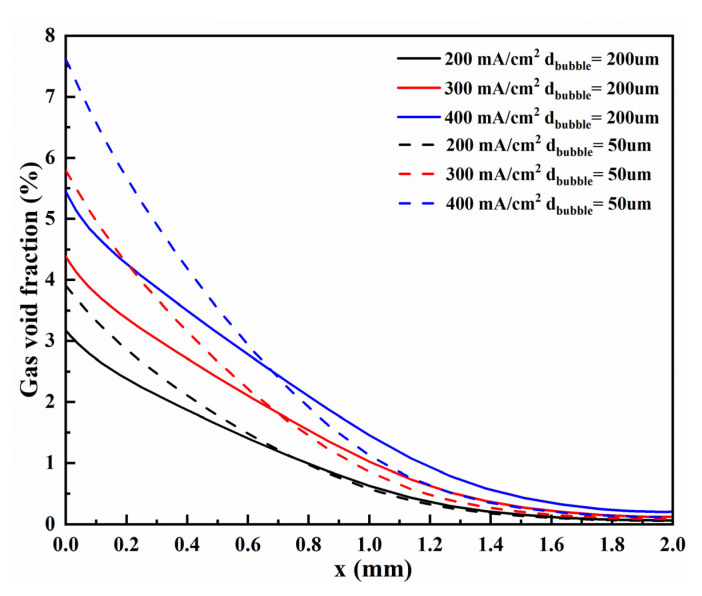
Gas void fraction curves of these two bubble diameters at the top of the H_2_ compartment under different current densities.

**Figure 6:**
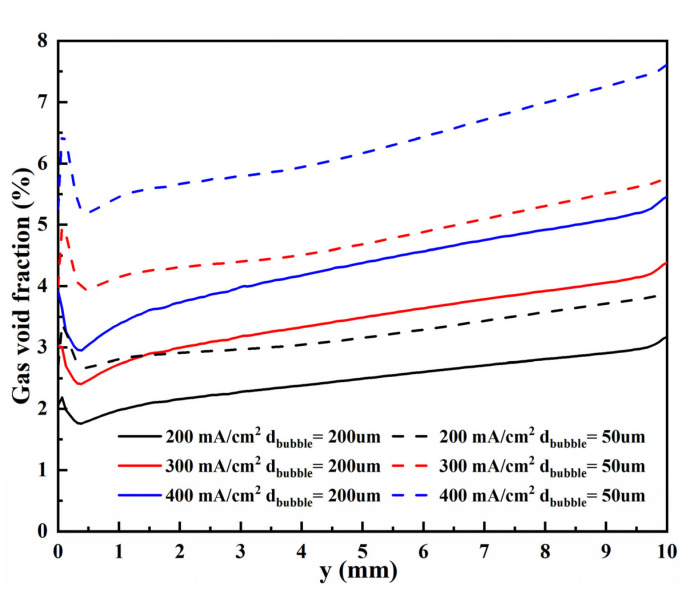
Gas void fraction curves of these two bubble diameters on vertical flat electrodes under different current densities.

Gas void fraction curves of these two bubble diameters at the top of the H_2_ compartment under different current densities are shown in Figure 5. Gas void fraction curves of these two bubble diameters on vertical flat electrodes under different current densities are shown in Figure 6. Bubbles diffuse along the x-direction, which is due to the different gas concentrations in the electrolyte. Meanwhile, the bubbles aggregate at the bottom of the cathode, increasing the void fraction and promoting the bubble diffusion along the y-direction. From a certain height of the electrode, the void fraction decreases along the y-direction, which is caused by the movement of bubbles away from the electrode.

For these two bubble sizes, the motion behavior of bubbles and the change of void fraction with current density are consistent. However, a smaller bubble size will lead to a higher void fraction. As shown in these figures, when the bubble diameter is 50 µm (flat-50), the void fraction on the electrode surface is higher than that of 200 µm (flat-200) at any current density. Moreover, as described in the literature [30], the thickness of the bubble layer will increase through larger bubbles, which is why the void fraction of bubble diameter of 200 µm will be slightly more significant after about 0.7 mm in Figure 5. Therefore, the trend that generating such tiny bubbles is beneficial to improving the efficiency of hydrogen production by electrolysis of water is consistent with the literature [47,48].

### 3.2. Micro-Nano Surface Electrode

#### 3.2.1. Polarization Curve

**Figure 7:**
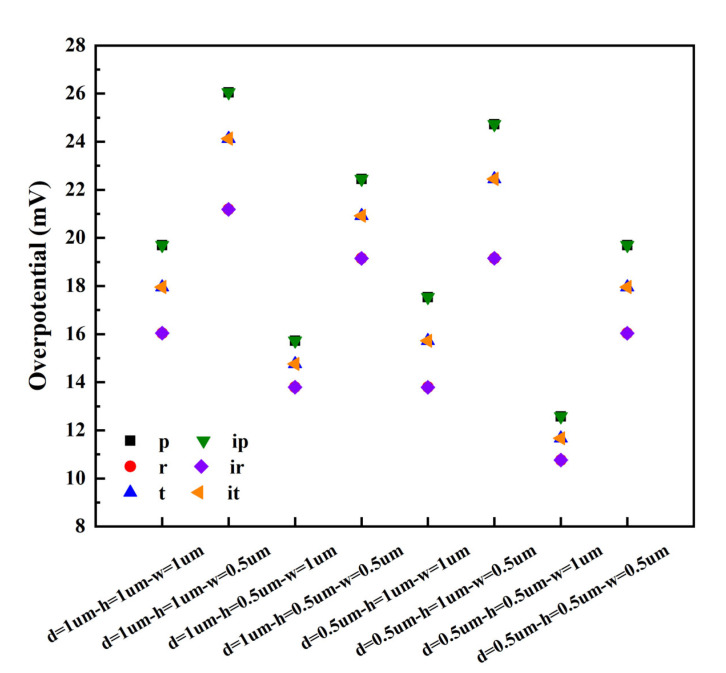
The overpotentials of micro-nano surface electrodes with different structure types and graphic parameters under the same current density of 10 mA cm^−2^.

The overpotentials of micro-nano surface electrodes with different structure types and graphic parameters under the same current density of 10 mA cm^−2^ are shown in Figure 7. It is seen that the overpotentials of p and ip, t and it, r, and ir are similar under the same graphic parameters, and the overpotentials of r and ir are the lowest, followed by t and it. Moreover, by sorting the overpotential from small to large, it is found that the corresponding graphic parameters of each structure are the same under the same ranking. For example, for all the structures, the overpotential is the smallest when the distance, height, and width are 0.5 µm, 0.5 µm, and 1 µm, respectively. However, the overpotentials of micro-nano surface electrodes with the same structure type and different graphic parameters are the same. For example, taking a rectangle as an example, their overpotentials are the same at graphic parameters of 1 µm, 1 µm, and 1 µm and 0.5 µm, 0.5 µm, and 0.5 µm. The differences between these micro-nano surface electrodes are reflected in the upward-inverted direction and micro-nano structural unit lengths.

**Figure 8:**
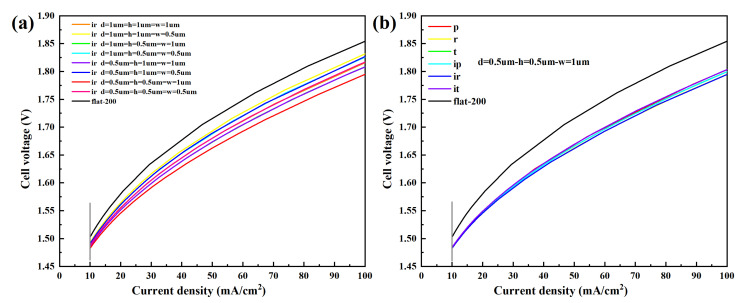
The polarization curves of the micro-nano surface and vertical flat electrodes. (**a**) The polarization curves of ir with different graphic parameters; (**b**) The polarization curves of six structures at the graphic parameters of 0.5 µm, 0.5 µm, and 1 µm.

The overpotentials of micro-nano surface electrodes with different structure types and graphic parameters and the vertical flat electrode under the same current density of 10 mA cm^−2^ are shown in Table 2. The polarization curves of the micro-nano surface and vertical flat electrodes are shown in Figure 8. Figure 8a shows the polarization curves of ir in different graphic parameters, and Figure 8b shows the polarization curves of six structures at the graphic parameters of 0.5 µm, 0.5 µm, and 1 µm. It can be noted that the cell voltage and cathode overpotential of all micro-nano surface electrodes are smaller than those of the vertical flat electrode. Compared with the vertical flat electrode, for the same graphic parameters (0.5 µm, 0.5 µm, and 1 µm), the overpotential of p, r, t, ip, ir, and it decreased by 59.44%, 65.31%, 62.40%, 59.44%, 65.31%, and 62.40%, respectively; for the same structure, the overpotentials of ir with different graphic parameters are reduced by 48.29%, 31.69%, 55.54%, 38.27%, 55.54%, 38.27%, 65.31%, and 48.29%, respectively.

The total length of micro-nano structural units and overpotential of each micro-nano surface electrode are shown in Table 3. The total length of micro-nano surface electrodes is larger than that of the vertical flat electrode. It can be concluded that the total length of micro-nano structural units affects the overpotential, and the larger the total length of micro-nano structural units, the lower the corresponding overpotential. Due to each group of structures (r and ir, p and ip, t and it) having the same total length of micro-nano structural units under the same graphic parameters, the overpotential is similar. Moreover, in these three groups of structures, the total length of micro-nano structural units corresponding to r and ir is more considerable, so the overpotential is the lowest. Due to the simulation model being a simplified 2D model of AWE, the length of micro-nano structural units is related to the contact area between the electrode and the electrolyte. Therefore, the increase in the length of micro-nano structural units means an increase in the contact area, which is helpful to increase the active site and reduce the cell voltage and overpotential, thus improving the electrolysis performance.

#### 3.2.2. Void Fraction

The gas void fraction of micro-nano surface electrodes under different structure types and graphic parameters are shown in Table 4. The average void fraction produced on the electrode surface was selected as a comparison. It is seen that the average void fraction produced by all micro-nano surface electrodes is greater than that of the vertical flat electrode. The structures of r and ir, p and ip, and t and it have a similar void fraction under the same graphic parameters, and the void fraction of r and ir is the largest, followed by t and it. In addition, according to the order of the average void fraction from large to small, it is found that the corresponding graphic parameters of each structure are the same under the same ranking. Compared with the vertical flat electrode, for the same graphic parameters (0.5 µm, 0.5 µm, and 1 µm), the average void fraction of p, r, t, ir, ip, and it increases by 50.60%, 54.53%, 52.91%, 51.46%, 54.75%, and 53.28%, respectively; for the same structure, taking ir as an example, the average void fraction of each graphic parameters increases by 45.10%, 37.68%, 48.21%, 39.96%, 48.76%, 40.66%, 54.75%, and 45.17%, respectively. Obviously, the changing trend of the average void fraction is consistent with the overpotential. If the total length of micro-nano structural units is the same, the corresponding void fraction is similar; if the total length of micro-nano structural units is larger, the corresponding void fraction is larger. Therefore, it can be concluded that the total length of micro-nano structural units also affects the void fraction. Meanwhile, with the increase in the total length of micro-nano structural units, the catalytic area increases, which leads to the increase in void fraction.

**Figure 9:**
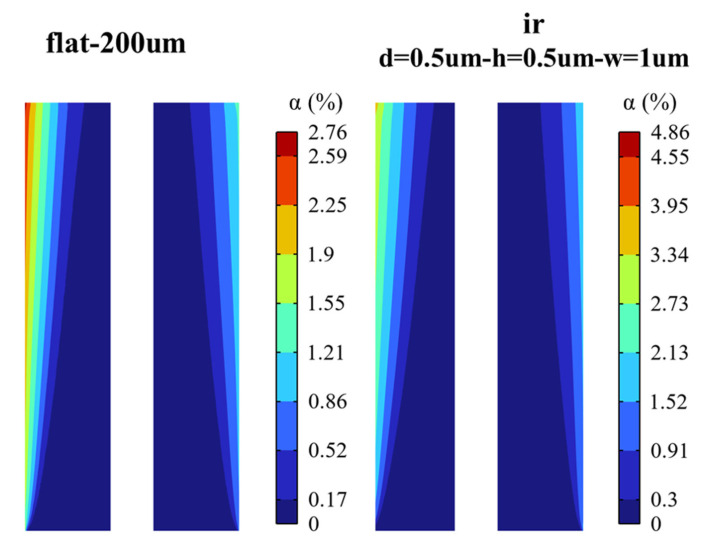
The gas void fraction distribution of micro-nano surface and vertical flat electrodes (U = 2 V).

Taking ir with distance, height, and width of 0.5 µm, 0.5 µm, and 1 µm as an example, the gas void fraction distribution of the micro-nano surface electrode and the vertical flat electrode is shown in Figure 9. It is seen that the gas distribution trend of the micro-nano surface electrode is consistent with that of the vertical flat electrode. However, the bubble layer produced by the micro-nano surface electrode is broader, and the local void fraction is higher.

**Figure 10:**
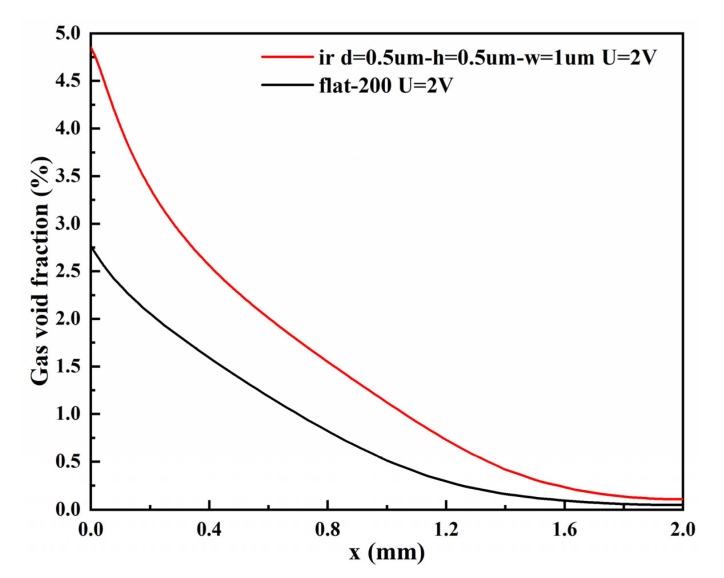
The gas void fraction curves of micro-nano surface and vertical flat electrodes at the top of the H_2_ compartment under the same voltage (U = 2 V).

**Figure 11:**
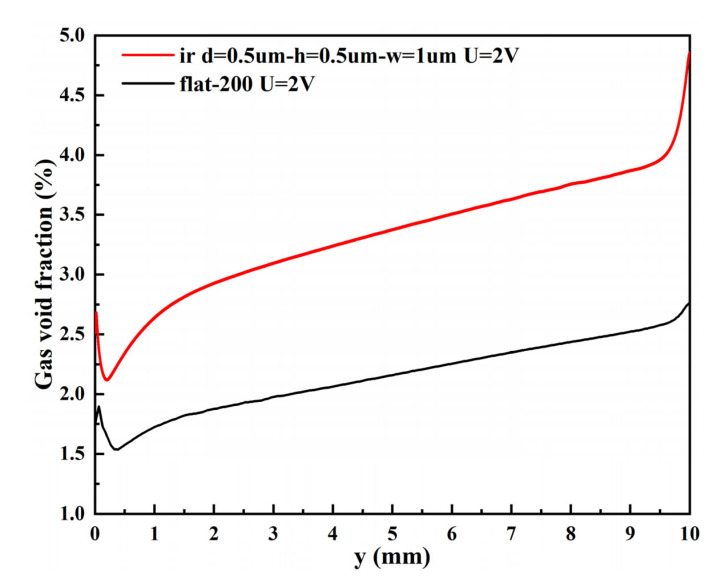
The gas void fraction curves of the micro-nano surface and vertical flat electrodes under the same voltage (U = 2 V).

The gas void fraction curves of the micro-nano surface electrode and the vertical flat electrode at the top of the H_2_ compartment under the same voltage are shown in Figure 10. The gas void fraction curves of the micro-nano surface electrode and the vertical flat electrode under the same voltage are shown in Figure 11. The void fraction of the micro-nano surface electrode in the x and y directions is more significant than that of the vertical flat electrode. Compared with the vertical flat electrode, the micro-nano surface electrode can produce more hydrogen and increase the void fraction due to the accumulation of bubbles, which leads to more turbulent diffusion and promotes the lateral penetration of bubbles. Furthermore, due to the limitation of H_2_ compartment height, bubbles cannot continue to diffuse upward and then accumulate, which leads to a significant increase in void fraction near the top of the micro-nano surface electrode compared with the vertical flat electrode.

Therefore, applying a micro-nano surface electrode helps increase hydrogen production and reduce cell voltage and overpotential, thus reducing energy consumption and improving the performance of the electrolyzer. Moreover, the effects of micro-nano surface electrodes with different structures and graphic parameters are different, so it is necessary to choose the appropriate structure type and graphic parameters according to the structural characteristics.

## 4. Conclusions

In this study, the influence of micro-nano surface electrodes on the electrolytic performance of the AWE was numerically investigated. The results that can be inferred from this research are as follows,

- The micro-nano surface electrode can significantly reduce the cell voltage and cathode overpotential and increase the gas void fraction. Among the six structures and graphic parameters set in this paper, when the distance, height, and width of each structure are 0.5 µm, 0.5 µm, and 1 µm, respectively, the cell voltage and cathode overpotential are the lowest, and the void fraction is the highest, and rectangle/inverted rectangle has the best effect, followed by trapezoid/inverted trapezoid.
- The bubble layers produced by the micro-nano surface and vertical flat electrode are parabolic distributed. However, compared with the vertical flat electrode, the void fraction produced by the micro-nano surface electrode is higher, and the bubble layer is thicker at any voltage.
- The total length of micro-nano structural units affects the cell voltage, cathode overpotential, and void fraction. With the increase in the total length of micro-nano structural units, the catalytic area increases, the cell voltage and overpotential decrease, and the void fraction increases. Taking rectangular structural units with a distance, height, and width of 0.5 µm, 0.5 µm, and 1 µm, respectively, as an example, the total length of the corresponding micro-nano surface electrode is three times that of the vertical flat electrode. However, compared with the vertical flat electrode, the cathode overpotential decreases by 65.31% and the void fraction increases by 54.53%.

## Figures and Tables

**Table 1:** Initial values of the 2D AWE model.

Symbol	Value	Unit	Description
R	8.314	J mol^−1^ K^−1^	Universal gas constant
F	96,485.3	C mol^−1^	Faraday constant
W_H2_	2	mm	Hydrogen compartment width
W_sep_	1	mm	Diaphragm width
W_O2_	2	mm	Oxygen compartment width
H_elec_	1	cm	Electrode height
W_r_	0.5, 1	µm	Structure width
H_r_	0.5, 1	µm	Structure height
D_r_	0.5, 1	µm	Structure distance
d_bubble_	50, 200	µm	Bubble diameter
T	70	°C	Operating temperature
p	1	atm	Pressure
i_0H2_	100	A m^−2^	Exchange current density, hydrogen oxidation
i_0O2_	1	A m^−2^	Exchange current density, oxygen reduction
M_H2O_	18	g mol^−1^	Water molar mass
M_H2_	2	g mol^−1^	Hydrogen molar mass
M_O2_	32	g mol^−1^	Oxygen molar mass
M_OH_	17	g mol^−1^	Hydroxide molar mass
c_KOH_	3	M	Electrolyte concentration
v_in_	0.2	m s^−1^	Average inlet velocity

**Table 2:** The overpotentials of micro-nano surface electrodes with different structure types and graphic parameters and the vertical flat electrode under the same current density of 10 mA cm^−2^.

Graphic Parameters (µm)			Overpotential (mV)						
Distance	Height	Width	p	r	t	ip	ir	it	flat-200
-	-	-	-	-	-	-		-	31.02
1	1	1	19.70	16.04	17.96	19.70	16.04	17.96	-
1	1	0.5	26.05	21.19	24.13	26.05	21.19	24.13	-
1	0.5	1	15.72	13.79	14.76	15.72	13.79	14.76	-
1	0.5	0.5	22.45	19.15	20.92	22.453	19.15	20.92	-
0.5	1	1	17.54	13.79	15.72	17.54	13.79	15.72	-
0.5	1	0.5	24.72	19.15	22.45	24.72	19.15	22.45	-
0.5	0.5	1	12.58	10.76	11.67	12.58	10.76	11.67	-
0.5	0.5	0.5	19.70	16.04	17.96	19.70	16.04	17.96	-

**Table 3:** The total length of micro-nano structural units and overpotential of each micro-nano surface electrode.

Graphic Parameters (µm)			Total Length of Micro-Nano Structure Units (mm)				Overpotential (mV)			
Distance	Height	Width	p/ip	r/ir	t/it	flat-200	p	r	t	flat-200
-	-	-	-	-	-	10	-		-	31.02
1	1	1	16.18	20.00	17.81	-	19.70	16.04	17.96	-
1	1	0.5	12.07	15.00	13.09	-	26.05	21.19	24.13	-
1	0.5	1	20.41	23.33	21.77	-	15.72	13.79	14.76	-
1	0.5	0.5	14.12	16.67	15.21	-	22.453	19.15	20.92	-
0.5	1	1	18.24	23.33	20.41	-	17.54	13.79	15.72	-
0.5	1	0.5	12.76	16.67	14.12	-	24.72	19.15	22.45	-
0.5	0.5	1	25.62	30.00	27.66	-	12.58	10.76	11.67	-
0.5	0.5	0.5	16.18	20.00	17.81	-	19.70	16.04	17.96	-

**Table 4:** The average void fraction of micro-nano surface electrodes under different structures and graphic parameters and the vertical flat electrode at the same voltage (U = 2 V).

Graphic Parameters (µm)			Average Void Fraction (%)						
Distance	Height	Width	p	r	t	ip	ir	it	flat-200
-	-	-	-	-	-	-	-	-	2.15
1	1	1	3.00	3.11	3.06	3.02	3.12	3.06	-
1	1	0.5	2.84	2.97	2.90	2.85	2.96	2.90	-
1	0.5	1	3.12	3.20	3.16	3.12	3.19	3.14	-
1	0.5	0.5	2.93	3.02	2.97	2.94	3.01	2.97	-
0.5	1	1	3.06	3.19	3.13	3.08	3.20	3.13	-
0.5	1	0.5	2.88	3.00	2.94	2.88	3.02	2.93	-
0.5	0.5	1	3.24	3.32	3.29	3.26	3.33	3.29	-
0.5	0.5	0.5	3.00	3.12	3.06	3.02	3.12	3.06	-

## Data Availability

Not applicable.

## Supplementary Materials

The following supporting information can be downloaded at: https://www.mdpi.com/article/10.3390/ma15144927/s1, Table S1: Grid study of the present work for flat-200 um model. Figure S1: Mesh generation for the used geometry. Figure S2: Study of grid independence. Figure S3: Comparison between the model of this study and the experimental results. Reference [8] are cited in the supplementary materials

## Nomenclature

$C_{\mu }$, $C_{\varepsilon ,1}$, $C_{\varepsilon ,2}$	constants in turbulence model, (-)	$\alpha_{c}$	cathodic charge transfer coefficient, (-)
$C_{d}$	drag coefficient, (-)	α	void fraction, (-)
$d_{b}$	bubble diameter, (m)	$\mu_{T}$	turbulent viscosity, (Pa s)
F	Faraday constant, (C mol^−1^)	µ	viscosity, (Pa s)
F_k_	volume force, (N m^−3^)	ρ	density, (kg m^−3^)
g	gravitational acceleration, (m s^−2^)	η	overpotential, (V)
j	current density, (A m^−2^)	σ	electrolyte conductivity, (S m^−1^)
j_0_	exchange current density, (A m^−2^)	ε	turbulent dissipation rate, (m^2^ s^−3^)
k	turbulent kinetic energy, (m^2^ s^−2^)	φ	electrical potential, (V)
M_g_	molar mass of the gas (kg mol^−1^)	τ	stress tensor, (N m^−2^)
n	number of transferred electrons, (-)	Subscripts	
p	pressure, (Pa)	l	liquid phase
R	universal gas constant, (J mol^−1^ K^−1^)	g	gas phase
Re	Reynolds number, (-)	Abbreviations	
u	velocity, (m s^−1^)	AWE	alkaline water electrolyzer
T	temperature, (°C)	KOH	potassium hydroxide
x, y	spatial coordinates, (m)	HER	hydrogen evolution reaction
Greek symbols		OER	oxygen evolution reaction
$\alpha_{a}$	anodic charge transfer coefficient, (-)	CFD	computational fluid dynamics
