# Supplementary material for: Numerical Study on Hydrodynamic Characteristics and Electrochemical Performance of Alkaline Water Electrolyzer by Micro-Nano Surface Electrode
Source: Materials (Basel). 2022 Jul 15;15(14):4927. doi: 10.3390/ma15144927 (PMC9316051; doi:10.3390/ma15144927)
Supplement: Supplementary file 1 [file materials-15-04927-s001.zip › materials-1779475-supplementary.pdf]

Supplementary Materials

Numerical study on hydrodynamic characteristics and electrochemical performance of alkaline water electrolyzer by micro-nano surface electrode

Ye Xia [1], Mengyu Gao [1,*], Jincheng Yu [1], Yang Si [1,2,*], Laijun Chen [1,2] and Shengwei Mei [1,2]

[1] New Energy (Photovoltaic) Industry Research Center, Qinghai University, Qinghai 810016, China; mygao16@fudan.edu.cn

[2] State Key Laboratory of Control and Simulation of Power System and Power Generation Equipment, Electric Machinery Department, Tsinghua University, Beijing 100084, China; siyang@qhu.edu.cn

* Correspondence: mygao16@fudan.edu.cn and siyang@qhu.edu.cn

1. Grid study and validation

In this study, a physics-controlled mesh was used to adopt the modules, and a triangular mesh was generated, as shown in Figure S1. Mesh elements were fine predefined on the electrolyte domain and extra fine on the electrodes and diaphragm boundaries to correctly handle the effects in these contours.

To ensure the quality of the simulation results, mesh independence tests were performed using different mesh types to determine whether a decrease in mesh size had a significant effect. Figure S2 shows the results of the grid sensitivity with five different meshes: coarse, normal, fine, finer, and extra fine. It can be noted that the maximum difference between these meshes is located at the velocity and gas void fraction peaks. According to the percentage error, as shown in Table S1, when the mesh types are coarse and normal, the velocity distribution has a noticeable deviation, and the error is relatively large. In contrast, when the mesh types are fine, finer, and extra fine, the simulated velocity distribution is independent of the mesh size, and the error is tiny. Moreover, the velocity distribution is obviously not improved when applied to finer and extra finer types. Therefore, fine mesh is used considering factors such as mesh independence, solution accuracy, and solution time.

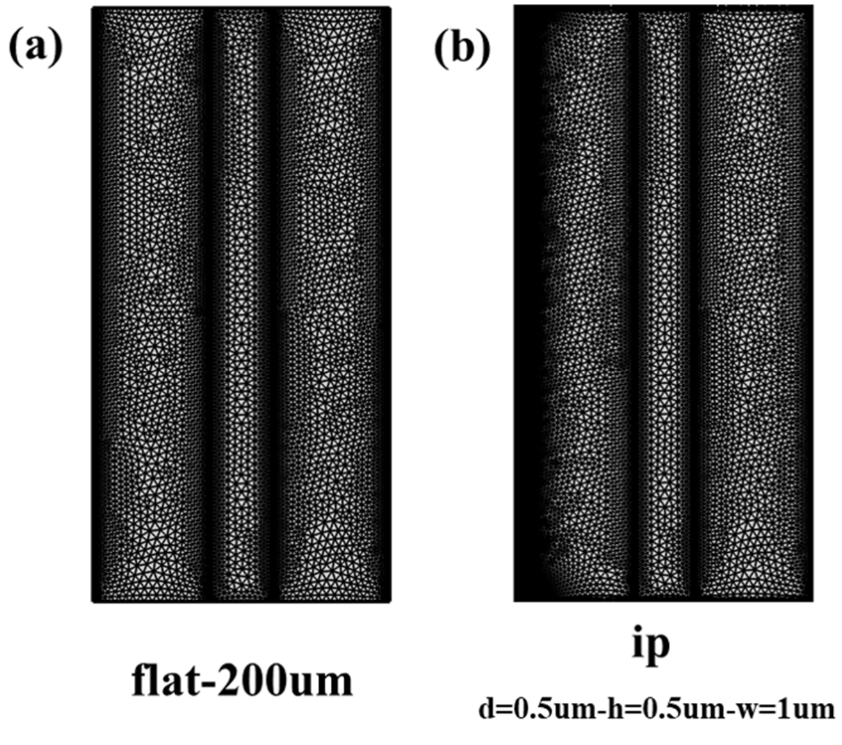

Figure S1. Mesh generation for the used geometry. (**a**) flat-200um. (**b**) ip (d=0.5um-h=0.5um-w=1um).

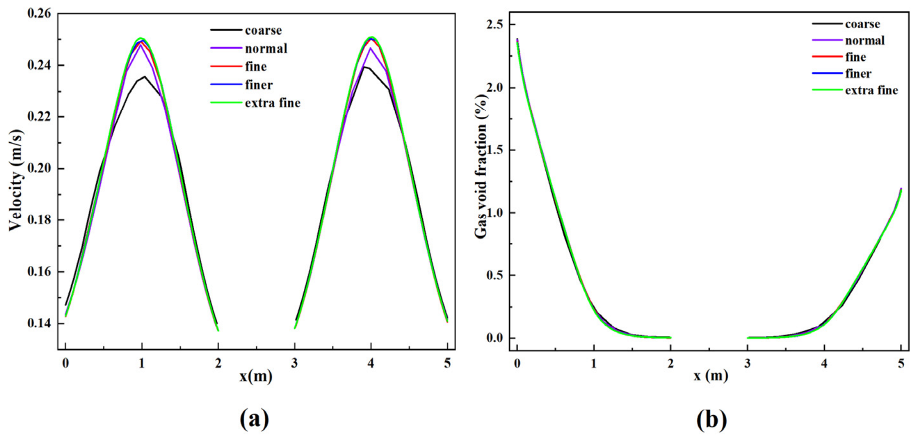

Figure S2. Study of mesh independence. (**a**) The effects of the mesh size on the velocity distribution. (**b**) The effects of the mesh size on the gas distribution.

Table S1. Grid study of the present work for the flat-200um model.

Mesh	Number of elements	Velocity (m/s)	Error (%)
coarse	4460	0.19718	1.29
normal	6624	0.19734	1.21
fine	18222	0.19917	0.28
finer	48088	0.19967	0.03
Extra finer	59564	0.19973	-

2. Model validation

Concerning the model validation, we referred to the theoretical value of Ni exchange current density given by Morales-Guio et al. [1], applied it to our numerical model, and carried out corresponding electrolysis experiments with Ni foam, as shown in Figure S3. It can be noted that the numerical results are in good agreement with the experimental results, and the error of the polarization curve is about 3%.

All electrochemical properties were investigated for the experiment on a CHI760E electrochemical workstation. A typical three-electrode configuration cell was used, in which Ni foam (2 cm × 1 cm × 0.5 mm) was the working electrode, Pt mesh slide (1 × 2 cm^2) was the counter electrode, and RHE was the reference electrode. The three-electrode configuration cell was placed in a 3.0 M KOH aqueous solution, and its effective geometric area was precisely controlled at 1 × 1 cm^2. The HER and OER polarization curves were collected at a scan rate of 1 mV s^{-1} via linear-sweep voltammetry (LSV). Before the measurement, the working solution was purged with N_2 for 40 min to remove dissolved oxygen with a flow rate of 150 mL/min. All the electrolysis was conducted at 70℃, and 90% IR correction was applied in all measurements.

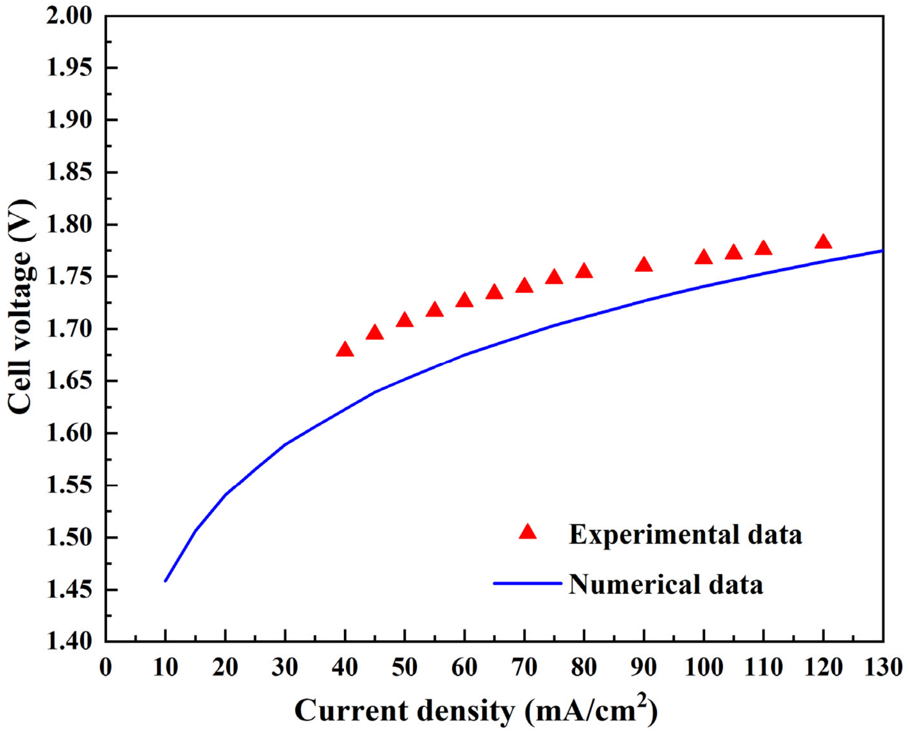

Figure S3. Comparison between the model of this study and the experimental results.

References

1. Morales-Guio, C.G.; Stern, L.A.; Hu, X. Nanostructured hydrotreating catalysts for electrochemical hydrogen evolution. *Chem. Soc. Rev.* **2014**, *43*, 6555–6569.
